# Sustainable development and ANN-based prediction of bio-waste-filled flax–pineapple–epoxy hybrid composites for enhanced mechanical performance

**DOI:** 10.1038/s41598-026-37015-x

**Published:** 2026-06-01

**Authors:** Sandeepkumar Gowda, Maruthi Prashanth B H, Ramesh S, Divijesh Puninchathaya P, Nidhin Raj A, Asif Iqbal Mulla, B Kiran Kumar, Priyaranjan Sharma, Gajanan Anne

**Affiliations:** 1 Department of Mechanical Engineering, Manipal University Jaipur, Dehmi Kalan, Jaipur, 303007 Rajasthan India; 2 Manipal Institute of Technology, Manipal Academy of Higher Education, Manipal, India; 3 Department of Mechanical Engineering, AGMR College of Engineering and Technology, Hubli, 581207 Karnataka India; 4 School of Computer Science and Engineering, RV University, Bangalore, 560059 India; 5 Department of Mechanical Engineering, Nitte (Deemed to be University), NMAM Institute of Technology (NMAMIT), Nitte, Karkalla, 574110 India; 6 Department of Mechanical Engineering, Adi Shankara Institute of Engineering & Technology, Ernakulam, 683574 Kerala India; 7 Department of Electronics & Communication Engineering, AGMR College of Engineering and Technology, Hubli, 581207 Karnataka India; 8 Department of Mechanical Engineering, Koneru Lakshmaiah Education Foundation, Vijayawada, 522302 Andhra Pradesh India

**Keywords:** Hybrid composite, Bio-waste fillers, Natural fibers, Fracture toughness, Impact strength, Artificial neural network, Engineering, Materials science

## Abstract

With the increasing demand for sustainable and cost-effective materials, natural fiber-reinforced composites are gaining traction among manufacturers and consumers. This study addresses the growing need for eco-friendly and structurally reliable composite materials. It focuses on the incorporation of bio-waste fillers into epoxy composites reinforced with flax and pineapple fibers, assessing their suitability for lightweight structural applications in automotive and construction sectors. Four fillers—coconut shell powder (CSP), teak wood dust (TWD), eggshell powder (ESP), and rice husk powder (RHP)—were added at a fixed 10 wt% to fabricate hybrid composites using a combination of hand layup and hot-pressing techniques. Mechanical properties such as tensile strength, impact resistance, interlaminar shear strength (ILSS), fracture toughness, and flexural strength were evaluated. Among all, CSP-reinforced composites showed superior mechanical performance, with enhancements ranging from 1.95% to 42.8% over other variants. Scanning Electron Microscopy (SEM) analysis revealed improved fiber–matrix bonding and minimal voids in CSP composites. In addition, an Artificial Neural Network (ANN) model was employed to predict mechanical properties with high accuracy: 95.85% (tensile), 83.9% (flexural), and 89.83% (impact). These results underscore the potential of using agricultural and industrial waste fillers in natural fiber composites for sustainable, high-performance applications in structural engineering.

## Introduction

 Agricultural waste contributes significantly to pollution and landfill overflows, negatively impacting rainwater infiltration and environmental health. While waste generation is unavoidable, materials such as natural fibers, shells, and husks can be effectively repurposed into bio-composites. These sustainable alternatives help lower production costs and reduce CO₂ emissions. However, inconsistencies in particle size, shape, and moisture content continue to pose challenges to achieving uniform composite properties and performance^1–4^. Petroleum-based plastics, though durable, are non-biodegradable, leading to persistent environmental pollution.

Saha et al.^5^ conducted an experimentally validated FEM-based micromechanical study on pineapple leaf fiber and particulate–reinforced polymer composites. Hybrid composites showed improved performance over conventional FRP, with increases of 10.16% and 26.36% in longitudinal and transverse Young’s moduli, and 9.91% and 26.17% improvements in in-plane and out-of-plane shear moduli, respectively, demonstrating the effectiveness of pineapple leaf micro-particulates as secondary reinforcements.

Saha et al.^6^ experimentally investigated bamboo fiber–reinforced biocomposites with different epoxy matrices, highlighting the strong influence of matrix type on performance. S1 composites showed the lowest density (1.02 g/cm³), highest bio-content (61.78%), and highest stiffness, while S2 composites exhibited superior tensile strength (144.76 MPa), lowest moisture absorption (4.49%), highest storage modulus (8.82 GPa), and glass transition temperature (111.72 °C). S3 composites achieved minimum void content (1.2%) and highest hardness, whereas S4 composites were the most cost-effective. The VIKOR-based MCDM approach identified S2 as the most suitable for automotive interiors, demonstrating the importance of matrix optimization despite challenges such as hydrophilicity and interfacial bonding.

Hayajneh et al.^7^ reviewed the effects of various natural fillers on the mechanical, thermal, and tribological properties of polymer matrices like polypropylene, polyethylene, PVC, and polyester. Their findings indicated that while tensile strength generally decreased with natural filler inclusion, flexural and tensile modulus typically improved. Impact strength varied depending on the intrinsic properties of each filler. This review provided valuable comparative insights for optimized filler selection in sustainable composite design.

Alaaeddin et al.^8^ comprehensively reviewed polyvinyl fluoride (PVF), emphasizing its exceptional mechanical, thermal, chemical, and optical properties. The study highlighted PVF’s applications (notably as Tedlar^®^), its polymerization methods, and manufacturing processes, alongside comparisons with similar polymers like PVDF, PTFE, and PVC. PVF was found to be highly suitable for advanced applications in medical devices, renewable energy, and nanotechnology, though further quantitative analysis was recommended.

Al-Oqla et al.^9^ investigated novel lignocellulosic grape fiber/polyethylene composites under harsh environmental conditions such as wet, alkaline, and sulfate exposure. The study identified optimal reinforcement conditions and examined the effects of fiber treatments. Results revealed significant improvements in tensile strength—especially for 30 wt% dry and 20 wt% water-soaked fibers. The highest modulus of elasticity (292.06 MPa) was observed at 40 wt% with oil-soaked fibers. SEM analysis confirmed enhanced fiber–matrix interactions. Due to their biodegradability, affordability, and environmental benefits, natural fibers are now widely used across various applications.

Hayajneh et al.^10^ examined natural fibers found in Jordan using ASTM-standard tests to evaluate physical and mechanical properties, including density, moisture content, tensile strength, and modulus. Their findings identified several local fibers with superior properties to globally popular alternatives, providing a regional database and effective selection criteria for sustainable development.

Fares and Al-Oqla^11^ reviewed the role of green polymers like cellulose, starch, and chitin in sustainable electronics. Their biodegradability and tunable degradation rates support advancements in organic transient bioelectronics. Innovations included cellulosic electroactive paper and biodegradable OLEDs, with starch-based composites showing potential in piezoelectric and thermoelectric systems. In this study, bio-waste fillers such as teak wood dust (TWD), coconut shell powder (CSP), rice husk powder (RHP), and eggshell powder (ESP) are integrated into natural fiber-reinforced composites. Coconut shells, a common agricultural by-product, are particularly useful in particulate form.

Sarki et al.^12^ reported improved tensile performance in epoxy composites with 20 wt% CSP reinforcement. Bledzki et al.^13^ showed CSP-reinforced polypropylene composites achieved the highest tensile strength, with further gains of 30% when treated with a maleic anhydride coupling agent. Ojha et al.^14^ found that wood apple shell fillers outperformed CSP at 15 wt% loading due to fewer voids, highlighting the importance of minimizing porosity.

Rice husks, while abundant and low-cost, are often underutilized. Rout and Satapathy^15^ found that hybrids of rice husk and glass fiber reduced tensile strength due to poor adhesion and sharp husk edges. Aridi et al.^16^ reported that rice husk composites showed increased voids under lubricant oil exposure but retained good flexural properties in seawater. Nourbakhsh et al.^17^ found that rice husk-polypropylene composites absorbed less water than bark-based alternatives due to higher cellulose content. Tran et al.^18^ demonstrated that alkali and silane treatments significantly improved interfacial bonding in rice husk composites, increasing strength by 20%.

Teak wood dust, a biodegradable by-product of the timber industry, is strong and cost-effective. Maruthi et al.^19^ incorporated TWD into abaca-pineapple-epoxy composites, achieving 3–35% mechanical improvements at 6 wt% loading. Chauhan et al.^20^ reported enhanced hardness, impact, and compressive strength in TWD-based epoxy/glass composites. Dinesh et al.^21^ observed superior tensile and flexural strength in jute composites reinforced with Padauk wood dust compared to Rosewood.

Eggshell powder (ESP), primarily composed of calcium carbonate, offers landfill reduction and biocompatibility. Aburpa et al.^22^ showed that 6 wt% ESP in jute-epoxy, coir -epoxy, sisal-epoxy composites enhanced tensile, flexural and impact strength. Ra Ji et al.^23^ found improved dispersion and bonding with 5 wt% ESP in eggshell particulate-epoxy composites. Al-Oqla et al.^24^ developed hybrid composites with ESP and date palm leaflets in a polypropylene matrix, achieving up to 26% and 11% increases in tensile and flexural modulus, respectively. According to Hayajneh et al.^25^, ESP-based polypropylene composites performed better than other composites strengthened by lemon leaves on the basis of improved interfacial adhesion.

Herlina et al.^26^ studied on Paederia foetida fiber–alumina (PFs–Al₂O₃) epoxy composites which showed that hybridizing natural fibers with ceramic particles can significantly enhance composite properties. An optimal PFs–Al₂O₃ balance (30% PFs–10% Al₂O₃) provided superior tensile, impact, and thermal performance due to improved interfacial bonding. Hybrid composites also exhibited lower water absorption and swelling than PF-only systems, indicating better dimensional stability. SEM analyses reported fiber pullout and controlled voids that contributed to mechanical behavior. Overall, PFs–Al₂O₃ hybrids outperform single-reinforcement composites and demonstrate strong potential for advanced natural fiber composites.

Sari et al.^27^ researched on polyester composites reinforced with Pandanus tectorius fibers (DPs) and rice husk powder (RHs) showed that agricultural waste fillers can enhance impact strength and thermal stability. Studies reported that higher DPs/RHs contents lead to improved performance, supported by SEM evidence of better fiber–matrix interaction. Overall, DPs/RHs hybrids offered promising potential for lightweight, sustainable structural and thermal-insulation applications.

Sari et al.^28^ reviewed recent research on biopolymers such as PLA, PBS, and PHA reinforced with natural fibers like kenaf, hemp, and jute. These fibers offered promising sustainable alternatives to synthetic composites. Improved fiber surface treatments and nanofillers have enhanced mechanical, thermal, and moisture-resistant properties. Although these biocomposites demonstrated strong potential for lightweight construction and automotive applications, issues related to moisture sensitivity and long-term durability still need to be addressed. Overall, current advancements highlighted both the potential and challenges in developing high-performance, sustainable biocomposites.

Kumar et al.^29^ evaluated hybrid bio-composite laminates for aerospace interior applications using experimental characterization and an advanced multi-criteria decision-making framework. Forty-eight laminate configurations were assessed for physio-mechanical performance and cost, and the optimal design was identified using a linear Diophantine uncertain linguistic fuzzy TODIM approach. The study found the [P–G–O] stacking sequence to be a low-density, cost-effective alternative to carbon fiber laminates, supporting sustainable material substitution in aerospace interiors.

Saha et al.^30^ integrated experimental studies with ensemble-based machine learning models (XGBoost and Random Forest) to predict impact strength and cost of hybrid polymer composites for automotive applications. The ML framework efficiently handled multiple design parameters and was coupled with FEM-based impact and crushing analyses, linking data-driven predictions with physical behavior. The study demonstrated the effectiveness of ML models for optimizing hybrid composite performance and cost.

Nashat and Al-Oqla^31^ used Adaptive Neuro-Fuzzy Inference System (ANFIS) to give an idea about the predictions of mechanical properties of carbon fiber reinforced syntactic thermoset composites. Their model was very close to experiments with the help of which the optimization of lightweight high strength materials was achieved. Al-Oqla and Al-Jarrah^32^ also introduced a five-tier ANFIS model that based solely on cellulose and moisture content inputs was able to forecast the cellulosic fibers mechanical behavior in a very precise manner.

Despite the fact that bio-waste fillers in the polymer composites have been studied several times, little research has been conducted on the aspect of incorporation of such fillers in the natural fiber-reinforced hybrid systems particularly on the use of flax and pineapple fibers in an epoxy base. No prior work has explored the combined effect of multiple bio-waste fillers such as coconut shell powder (CSP), teak wood dust (TWD), eggshell powder (ESP), and rice husk powder (RHP)—within such a hybrid natural fiber composite. To bridge this gap, the present study develops a novel flax-pineapple fiber reinforced epoxy composite, incorporating these bio-waste fillers to evaluate their collective influence on mechanical performance. A two-step process hand layup and hot pressing was employed to fabricate the composites, which were then mechanically tested for tensile, impact, hardness, flexural, and interlaminar shear strength (ILSS). Scanning Electron Microscopy (SEM) analysis was carried out to investigate fracture morphology and filler-matrix interaction. Furthermore, a Keras Sequential Model was implemented to predict key mechanical properties, introducing an AI-driven approach to composite behavior prediction. This unique integration of multiple natural fibers, diverse bio-waste fillers, and machine learning techniques demonstrates the novelty of the study and its contribution toward sustainable, high-performance composite development.

## Experimental details

### Materials

Pineapple and flax leaf fibers were obtained from Go-Green Products, a supplier specializing in natural fibers based in Bangalore, India. Teak wood dust was collected from a nearby wood processing facility, while eggshells and coconut shells were gathered from local sources. Rice husk was acquired from a local rice mill. For composite fabrication, LY556 epoxy and HY951 hardener were acquired from Shri Durga Chemicals, Mangalore, and prepared using a 10:1 mixing ratio. Tables 1 and 2 summarize the physical, chemical, and mechanical characteristics of flax and pineapple fibers. Table 3 provides the chemical and physical properties of the bio-fillers—coconut shell powder (CSP), rice husk powder (RHP), teak wood dust (TWD), and eggshell powder (ESP). The physical and mechanical properties of the epoxy resin system are listed in Table 4.

**Table 1:** Chemical, physical, and mechanical properties of flax fiber^33^.

Chemical properties		Physical & mechanical properties	
Cellulose (%)	60–80	Density (g/cm^3^)	1.5
Hemicellulose (%)	12–20	Diameter of fiber bundle (mm)	0.06
Lignin (%)	2–5	Tensile strength (MPa)	1339–1800
Pectin (%)	2–12	Youngs modulus (GPa)	39–78
Waxes (%)	2–5	Elongation at break load (%)	2–3.5
		Thermal conductivity (W/m·K)	0.04–0.06

**Table 2:** Physical, mechanical, and chemical properties of pineapple leaf fiber^33^.

Physical & mechanical properties		Chemical properties	
Density (g/cm^3^)	1.4–1.6	Cellulose (%)	70–82
Diameter of fiber bundle (mm)	0.08	Hemicellulose (%)	6–12
Tensile strength (MPa)	413–1627	Lignin (%)	5–12
Youngs modulus (GPa)	2–6	Pectin (%)	1–1.2
Elongation at break load (%)	1–4	Waxes (%)	3–3.3
		Thermal conductivity (W/m·K)	0.034–0.06

**Table 3:** Physical and chemical properties of various natural fillers^34,35^.

Type of natural filler	Physical property	Chemical properties					
	Density (g/cm^3^)	Cellulose (%)	Hemicellulose (%)	Lignin (%)	Calcium carbonate (%)	Calcium phosphate (%)	Other minerals (%)
Coconut shell powder	0.7	36–43	41–45	0.15–0.25	–	–	–
Rice husk powder	0.12	25–35	18–21	26–31	–	–	–
Teak wood dust	0.65	40–50	20–30	20–30	–	–	–
Egg shell powder	2.25	–	–	–	93–95	1	4–6

**Table 4:** Physical and mechanical properties of epoxy resin^36^.

Density (g/m^3^)	0.65
Tensile strength (MPa)	80
Youngs modulus (GPa)	11.5
Thermal conductivity (W/m·K)	0.2

### Processing of natural fillers

Each natural filler—coconut shell powder (CSP), rice husk powder (RHP), teak wood dust (TWD), and eggshell powder (ESP) were separately soaked in water and manually stirred for about five minutes to eliminate surface impurities such as dirt and mud. The fillers, once cleaned, were kept in an oven at 40 °C for an hour to dry. After drying, they were sieved to collect particles smaller than 300 μm in size.

### Chemical processing of flax and pineapple leaf fibers

**Fig. 1:**
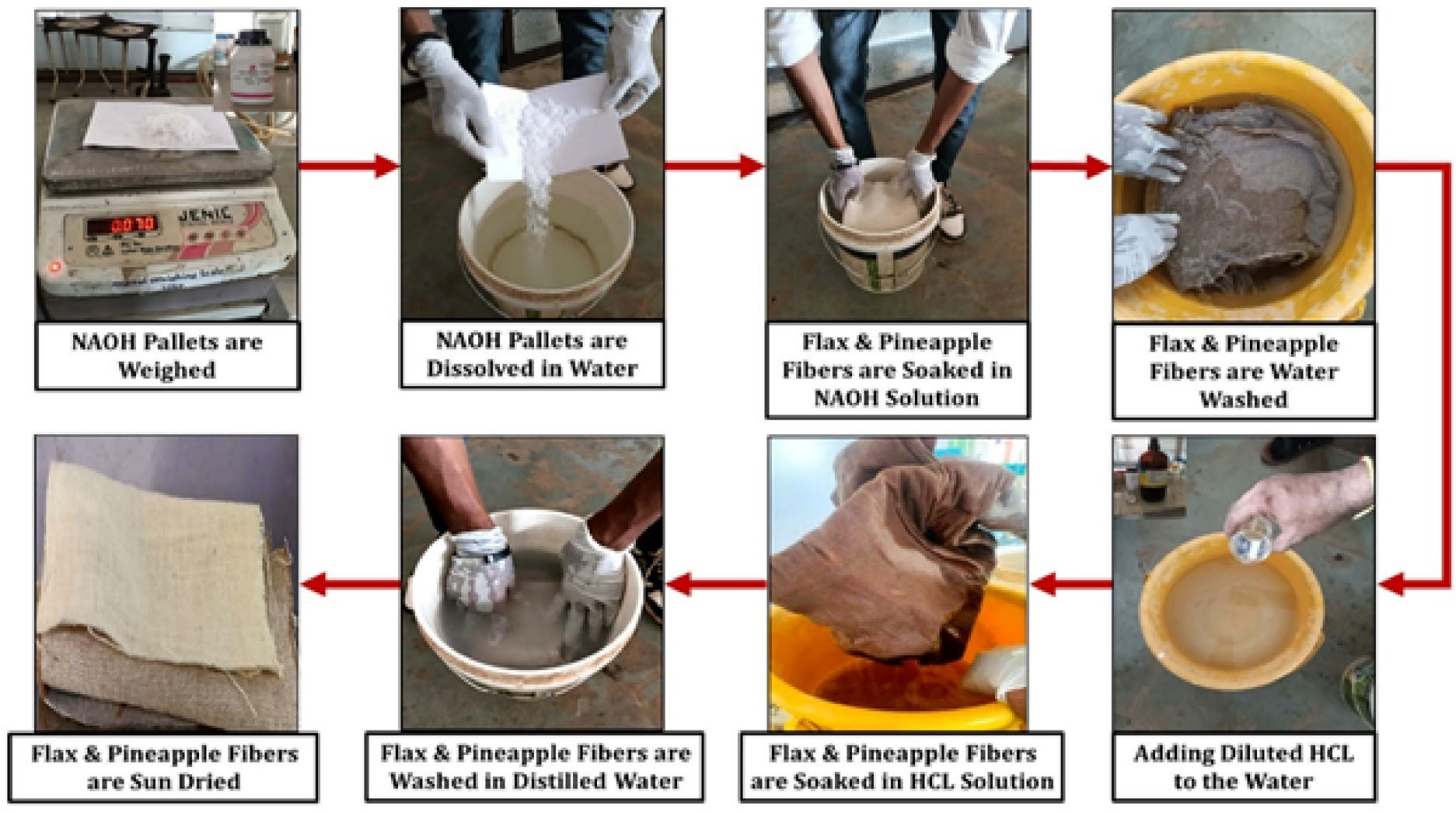
Stepwise chemical treatment procedure for flax and pineapple leaf fibers.

For fiber treatment, NaOH pellets were dissolved in 1 L of water to make a 5 wt% solution, as described in^37^. The flax and pineapple leaf fibers were soaked in the alkaline solution for one hour, followed by rinsing with a 5 wt% hydrochloric acid (HCl) solution—prepared by diluting HCl in 1 L of water—to neutralize any remaining alkalinity. Following this neutralization step, the fibers were rinsed thoroughly with clean water and then left to dry in the sunlight for about six hours to eliminate any moisture. The chemical treatment procedure is illustrated in Fig. 1.

### Fabrication of hybrid composite

The quantities of flax fiber, pineapple leaf fiber, individual bio-waste fillers (processed separately), and epoxy resin were calculated using the rule of mixtures. The basic formulas for the rule of mixtures are presented in Eq. (1), Eq. (2), Eq. (3), Eq. (4), and Eq. (5). Pre-impregnated fiber mats (pre-pregs) were prepared by coating the flax and pineapple leaf fibers with a measured amount of epoxy resin. Afterward, the samples underwent semi-curing at 60 °C in an oven for nearly one hour. After semi-curing, the pre-pregs were arranged in defined orientations. Each natural filler coconut shell powder (CSP), rice husk powder (RHP), teak wood dust (TWD), and eggshell powder (ESP) was added at 10 wt% to the epoxy resin, mixed thoroughly, and applied manually between the flax and pineapple fiber mats using the hand lay-up technique. The entire laminate was then pressed in a portable hot-pressing machine at 120 °C and 1 MPa pressure to enhance bonding and reduce voids formed during lay-up.

**Fig. 2:**
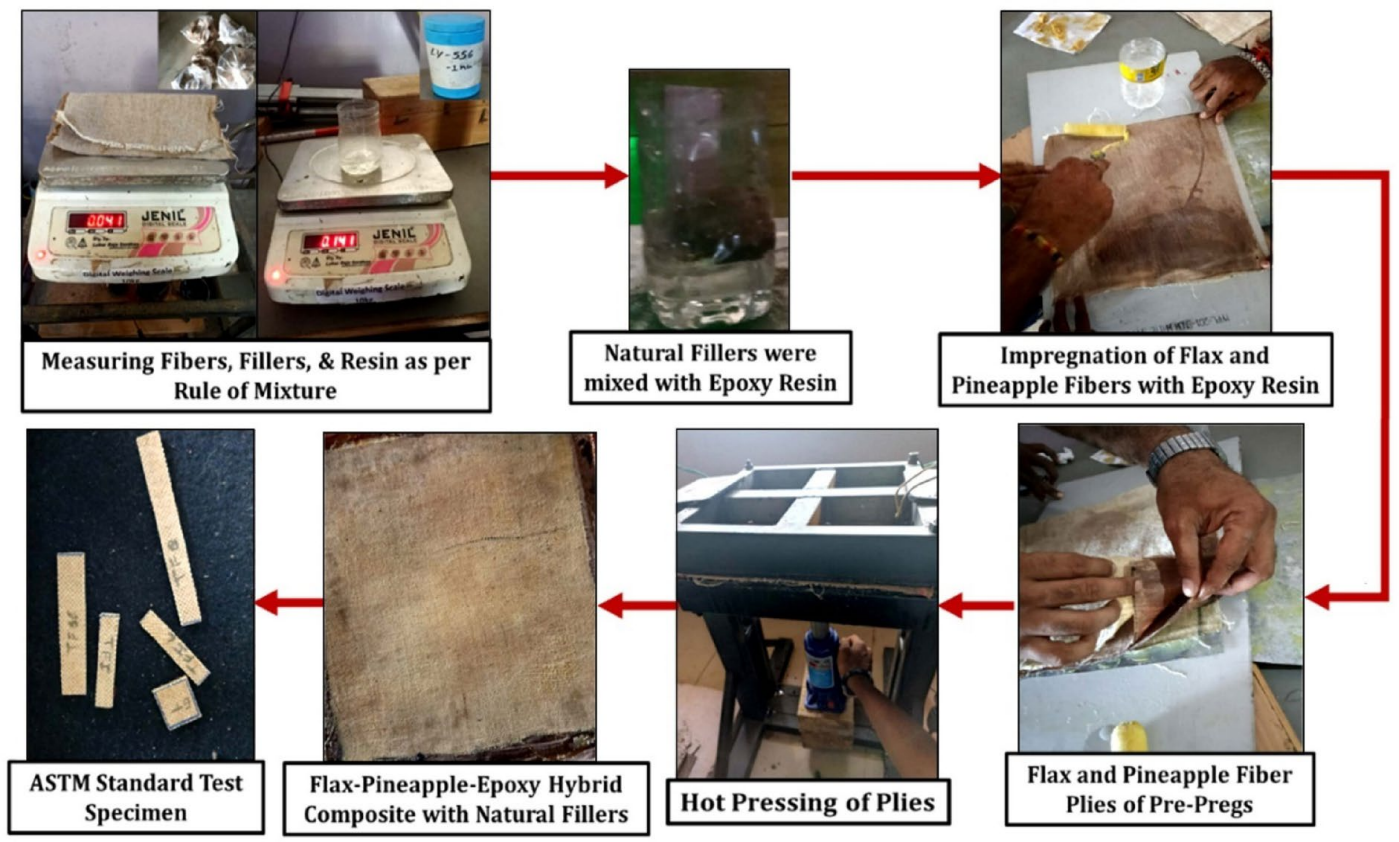
Sequential stages involved the fabrication of flax-pineapple-epoxy hybrid composites incorporating various natural bio-waste fillers.

**Fig. 3:**
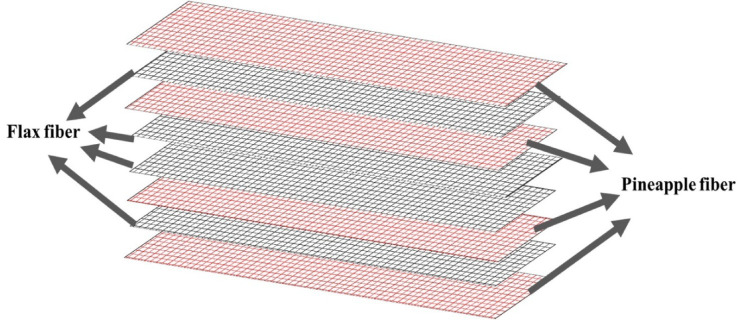
Stacking sequence of flax- pineapple fiber composite.

For every composite variation, four specimens were fabricated in accordance with ASTM testing standards. A coding system was used to identify each composite type: “CSP” for coconut shell powder, “RHP” for rice husk powder, “TWD” for teak wood dust, “ESP” for eggshell powder, and “WF” for the control sample without any filler. Each formulation contained 10 wt% of its respective filler blended into the epoxy matrix. Figure 2 shows the entire processes of fabrication or preparation of the flax-pineapple fiber reinforced epoxy composite prepared with bio-wastes fillers. The precise ratio of the weight of flax fiber, pineapple fiber, epoxy resin and fillers is tabulated in Table 5. Figure 3 shows stacking sequence, Pineapple-Flex-Pineapple-Flex- Flex- Pineapple-Flex-Pineapple fiber layers were used in this work, Filler-rich epoxy layers applied between each ply, Thickness per ply: 0.35–0.40 mm, Final cure: 120 °C, 1 MPa 8-ply laminate, [0/90] woven flax mat alternating with [0/90] pineapple mat. Table 6 presents the volume fraction of abaca fiber, bagasse fiber, and polyester resin.

1 $$\:{\rho\:}_{c}=\frac{1}{\frac{Wt.\%\:of\:Flax\:fiber}{{\rho\:}_{F}\:}+\:\frac{Wt.\%\:of\:Pineapple\:fiber}{{\rho\:}_{P}}+\:\frac{Wt.\%\:of\:Epoxy\:resin}{{\rho\:}_{E}}+\frac{Wt.\%\:of\:Each\:Filler\:}{{\rho\:}_{Fi}}}$$

2 $$\:{V}_{F}=\frac{Wt.\%\:of\:Flax\:fiber\times\:{\rho\:}_{c}}{{\rho\:}_{F}}$$

3 $$\:{V}_{P}=\frac{Wt.\%\:of\:Pineapple\:fiber\times\:{\rho\:}_{c}}{{\rho\:}_{P}}$$

4 $$\:{V}_{E}=\frac{Wt.\%\:of\:Epoxy\:Resin\times\:{\rho\:}_{c}}{{\rho\:}_{E}}$$

5 $$\:{V}_{Fi}=\frac{Wt.\%\:of\:Each\:Filler\times\:{\rho\:}_{c}}{{\rho\:}_{Fi}}$$

Where $\:{\rho\:}_{c},\:{\rho\:}_{F},\:{\rho\:}_{P},\:{\rho\:}_{E}{,\:\rho\:}_{Fi}$ are the density of composite configuration, flax fiber, pineapple fiber, epoxy resin and each filler at a time respectively. $\:{V}_{F},\:{V}_{P},\:{V}_{E}{,\:V}_{Fi}$ are the volume fraction of flax fiber, pineapple fiber, epoxy resin and each filler at a time respectively.

**Table 5:** Composite composition detailing the weight percentages of different fibers.

Sample name	Flax fiber wt%	Pineapple leaf fiber wt%	Natural fillers by wt%				Epoxy resin and hardener wt%	Experimental density(g/cm^3^)	Void content in %
			CSP	TWD	ESP	RHP			
Coconut filler (CF)	20	20	10	-	-	-	50	1.28	2.31
Teak filler (TF)			-	10	-	-	50	1.18	2.35
Eggshell filler (EF)			-	-	10	-	50	1.23	2.35
Rice-husk filler (RF)			-	-	-	10	50	1.26	2.21
Without filler (WF)	20	20	-	-	-	-	60	1.31	2.42

**Table 6:** Volume fraction of fibers and resin for different composite composition.

Sl. no.	Volume fraction % of flax fiber	Volume fraction % of pineapple leaf fiber	Volume fraction % of epoxy resin	Volume fraction % of each filler					Specimen sample name
				CSP	TWD	ESP	RHP	WF	
1	11.31	11.31	65.26	12.12	–	–	–	–	CF
2	11.21	11.21	64.66	–	12.93	–	–	–	TF
3	12.34	12.34	71.20	–	–	4.11	–	–	EF
4	7.13	7.13	41.15	–	–	–	44.58	–	RF
5	11.21	11.21	64.66	–	–	–	–	12.93	WF

## Mechanical testing of composites

### Tensile test

Tension testing (or Tensile Testing), is a routine and well-established technique applied to quantify the mechanical response of a material to uniaxial traction. It gives vital data on ultimate tensile strength (UTS) of material, tensile modulus (E) and elongation at break; all of which are vital objects of determining structural structures and appropriateness to incorporate in engineering schemes. The tensile testing in the present research was determined in ASTM D3039^38^ which is the standard amount of measurement of tensile properties of fibre reinforced polymer matrix composites which use high-strength fibres. This will make results as reliable and comparable to the available literature. To have increased reliability associated with identical tests, the test specimens were accurately prepared with a standard dimension of 250 mm in length, 25 mm in width and 3 mm in thickness as per the specified standard. Four replicates were also made to test each kind of sample and to give a repeatability and statistical validity of the properties measured. The test was done on an Instron 1195 Universal Testing Machine, which was fitted with a 10-ton load cell and could provide a high level of accuracy during both the load and the elongation tests. The samples were held and a constant increasing tensile load was applied till failure where the applied force and the resultant elongation of the sample was continuously recorded by the machine.

Based on the gathered load-displacement models, some main mechanical characteristics were determined, including the ultimate tensile strength, which is the maximum stress the material can sustain before breaking, the tensile modulus, which is the steepness of the initial straight-line portion of the stress–strain curve and represents the material’s stiffness, and the elongation at break, which is the amount a material elongates before failure and serves as an indicator of ductility.

These properties are very important in the study of the behaviour of the composite when loaded in tension and also in the comparison of various reinforcement schemes or formulations. The findings can be used to evaluate the performance of the developed composite materials for potential structural or operational applications, and the tensile stress was calculated using Eq. (6).

6 $$\:\sigma\:=\:\frac{F}{A}\:$$

Where the tensile strength is represented by σ in N/mm², the maximum load before failure by F in newtons (N), and the cross-sectional area by A in mm², the tensile modulus E is determined using Eq. (7). The corresponding tensile strain, denoted by ε, should be clearly indicated in diagram (e).

7 $$\:E=\:\frac{\sigma\:}{\epsilon\:}\:$$

### Flexural test

The flexural testing was conducted to determine the bending behaviour of the reinforced composite materials as developed using flax-pineapple fibers hybrid. The tests conformed to ASTM D790^39^ the standard which provides the method of measuring the flexural characteristics of the unreinforced, and reinforced plastics studies based on the three-point bending technique. The test engineering was performed with the Instron 1195 universal testing machine in which the load cell is 10 kN which would provide a well-defined application of load and measure of displacement. Using this arrangement, a measured response of the material to bending stresses can be assessed precisely. The specimen to be tested was tried on dimensions of 127 mm in length, 12.7 mm in width and 3 mm in thickness following the required requirements of ASTM. The three point bending test configuration was done with a span length of 50 mm. Replicate tests were carried out to provide the statistical reliability and reproducibility of the tests to provide a number of four replicate specimens of each composite variant. This test facilitates in knowing the largest flexure stress (sigma b) that mineral composite material can stand to failure which serves all the needed information on the load capacity and the stiffness of the material when under bending. The flexural strength was determined by the following formula, as given in Eq. (8).

8 $$\:{\sigma\:}_{b}=\:\frac{3FL}{2b{t}^{2}}$$

Where σ_b_ indicates the flexural strength in N/mm², ‘F’ is the maximum load at failure in newtons (N), ‘L’ is the span length of the specimen in millimetres (mm), while ‘b’ and ‘t’ represent the specimen’s width and thickness in millimeters (mm), respectively.

The flexural modulus of the composite specimen is obtained from the formula given in Eq. (9).

9 $$\:{E}_{b}=\:\frac{m{L}^{3}}{4b{t}^{2}}$$

### Interlaminar shear strength (ILSS) test

The ASTM D2344/D2344M-16^40^ ILSS test measures how well layers in a composite resist shear force. Flax-Pineapple leaf fiber hybrid composite specimens, 64 mm × 10 mm × 2.5 mm, are conditioned at 23 °C and 50% humidity for over 40 h. A Universal Testing Machine (UTM) with a three-point bending fixture and a 10 mm support span (four times specimen thickness) is used, featuring 5 mm radius on loading nose and supports. Load is applied at 1 mm/min, causing failure within 1 to 2 min.

### Impact test

The effect of resistance to impact was determined through Izod impact testing using Zwick-Roell impact testing machine to determine the impact resistance of the developed flax-pineapple fiber hybrid composites. Tests were conducted in accordance with ASTM D256^41^ standard, which establishes the Izod pendulum as a procedure for measuring the impact strength of plastic and polymer-composite materials. Test specimen was carefully made with uniform sizes of 64 mm by 12.7 mm by 3 mm. All specimens were machined to have a V-notch of 2.54 mm deep and 45 notch angles, which is the ASTM specifications. The notch serves a purpose of a stress concentrator to guarantee starting the fracture at a specific point to facilitate reproducible and comparable sample-to-sample results. In the results, the specimen was tested vertically on the impact tester, notch side facing towards the impact direction. The Zwick-Roell machine pendulum was dropped a known distance thus impacting the unbounded end of the specimen to cause fracture to occur. The machine made an automatic recording of impact energy absorbed by the specimen in fracture. The samples of each composite configuration under test were performed on four replicate specimens in order to guarantee repeatability and statistical significance. The impact strength (Izod impact value) was determined by applying the amount of energy that was received (in joules) using Eq. (10).

10 $$\:Impact\:Strength=\:\frac{{E}_{i}}{t}$$

where ‘$\:{E}_{i}$’ represents the impact energy (J), and ‘t’ indicates the specimen thickness (mm), respectively. This test is used to assess the toughness and ability of the material to withstand sudden or shock loading.

### Single edge notch bending (SENB) test

Mode-I criteria were used to measure the fracture toughness of the developed flax-pineapple fiber hybrid composites in order to estimate how resistant the material was to the propagation of crack. The test was carried out based on ASTM D5045- 14^42^ standard that describes the procedure of obtaining plane-strain fracture toughness (KIC) of polymer and polymer-based composite materials with the use of Single-Edge Notch Bending (SENB) specimens.

The test bars were made such that the dimensions were precise and were 32 mm in length, 12.5 mm in width and 3 mm thick. The pre-crack introduced was sharp because a mid-span notch was cut on the specimen to have a crack length-to-width (a/W) ratio of 0.5, per the requirements within the standard to be able to evaluate the fracture toughness. Such a setup encourages the initiation and growth of cracks under a carefully set bending stress to easy computation of KIC.

Tests on fracture were performed with a Universal Testing Machine (Instron) having necessary load cell capacity and three-point bending fixture. The machine was used with a gradually increasing load at a reined-up rate until the specimen fracture progressively. Each specimen had the critical load (Pc) at which the propagation of cracks in the material was observed. To ensure repeatability and statistical significance, four replicate specimens were tested for each composite configuration.

### Machine learning model

ANN or artificial neural network is a brain-based model of machine learning. It is a group of interconnected nodes (neurons) layered that can potentially learn complicated non-linear connections between data. ANNs find a great utilization in materials science where they are used to learn the mechanical, thermal or other properties of materials that are given as an input feature, such as fiber content, diameter, or chemical composition. Nashat Nawafleh and Faris M Al-Oqla^43^ presented a new comparative hybrid Particle Swarm Optimization-Artificial Neural Network (PSO-ANN) model to precisely foretell the mechanical features of natural fibers, the issue of intricacy and unpredictability of bio-fiber characterization. With the modification of non-linear activation functions, the ANN model uses important input factors including cellulose content, microfibrillar angle and fiber diameter to predict the values of tensile strength and Youngs modulus. The model saves a lot of time in selecting the right material to make green biomaterials because it lessens the need to conduct large-scale experiments. The high accuracy and consistency of the model were confirmed by means of testing against experimental findings. This ANN-based methodology manifests a quality instrument in optimization of natural fiber-reinforced composite facilitating speedier, more economical and green use of resources in the development of the materials. An integrated artificial intelligent (AI), which entails fuzzy clustering and stacked ensemble model, approach to predict mechanical properties of lignocellulosic fibers (that is expected to be a vital step in the direction of the creation of successful green biomaterials) was presented by Rami Al-Jarrah, and Faris M AL-Oqla^44^. The stacked method, although not a pure ANN model, has a high probability of accommodating several machine learning models, and possibly ANN, to enhance the accuracy of predictions. A thorough dataset was derived based on earlier research and has been used to train the model with the main fiber characteristics, height, diameter, and microfibrillar angle. The unseen data were used to check the predictions of ultimate tensile strength and elongation to break and showed high accuracy of predictions, with tolerable margins of errors. The method works by amalgamating fuzzy clustering and a stacked learning framework to manage the heterogeneity in the nature of natural fiber properties to minimize the ambiguity and enhance the reliability. Although not dedicated to ANN, the method indicates how AI-based models, such as neural networks as part of an ensemble of models, can assist material experts to maximize fiber choice in bio-composites with consideration of sustainability.

**Fig. 4:**
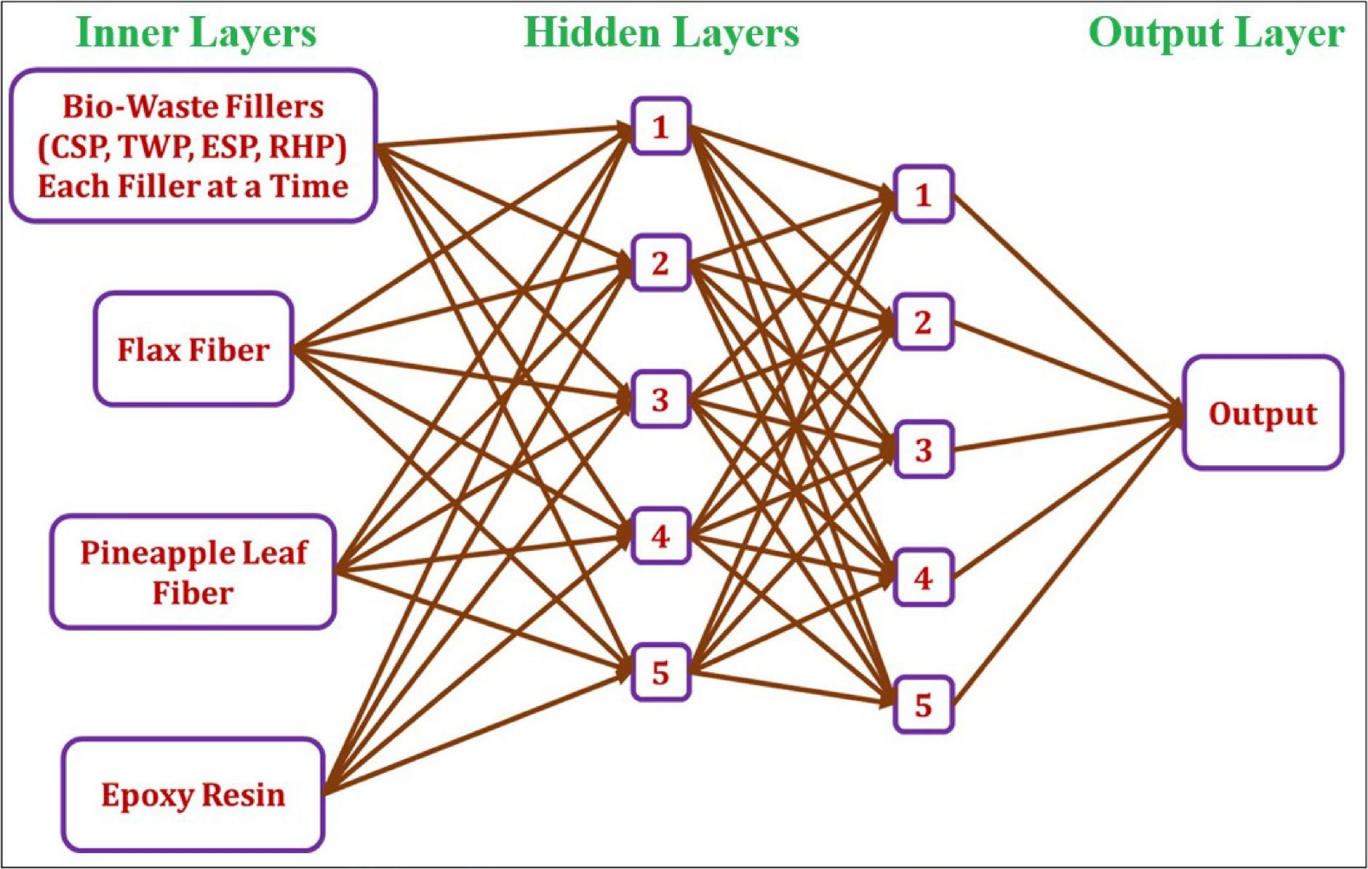
Schematic diagram of the TensorFlow sequential model.

To carry out this research, a Sequential Model (SM) based on TensorFlow was deployed to forecast three parameters: impact strength (IS), flexural strength (FS) and tensile strength (TS) of composite materials using variables, which are the percentage of composition of the constituents of the composite material(s). As shown in Fig. 4, the model follows a typical artificial neural network (ANN) structure, where data flows through a series of layers sequentially until the final output is generated. This ANN was developed and trained specifically for estimating the mechanical behavior of the composites based on the input formulation.

The model is evaluated using five distinct composite material configurations to predict tensile, flexural, and impact strengths individually:

1. Coconut shell powder, epoxy, flax fiber, and pineapple leaf fiber.
2. Teak wood dust powder, epoxy, flax fiber, and pineapple leaf fiber.
3. Eggshell powder, epoxy, flax fiber, and pineapple leaf fiber.
4. Rice husk powder, epoxy, flax fiber, and pineapple leaf fiber.
5. Epoxy, flax fiber, and pineapple leaf fiber.

To begin, the program generates 20 random sets of weight percentages for the four material components using the Dirichlet distribution, ensuring each combination totals 100%. Table 7 shows the datasets used for ANN prediction. The mechanical characteristics—impact strength, flexural strength, and tensile strength—are then estimated using the corresponding weighted-sum relations given by Eqs. (11), (12), and (13), each derived from the individual modulus values of the constituent materials. The resulting dataset is split into training (80%) and testing (20%) subsets, with the input data standardized using the Standard Scaler from the scikit-learn library.

11 $$\:TS=\sum\:_{i=1}^{4}\left(\frac{{W}_{i}\times\:{TS}_{i}}{100}\right)$$

12 $$\:FS=\sum\:_{i=1}^{4}\left(\frac{{W}_{i}\times\:{FS}_{i}}{100}\right)$$

13 $$\:IS=\sum\:_{i=1}^{4}\left(\frac{{W}_{i}\times\:{IS}_{i}}{100}\right)$$

Where, W_i_ denotes the weight% of the i^th^ material in the composite. The term TS_i_ refers to the tensile strength of the respective material, FS_i_ represents its flexural strength, and IS_i_ corresponds to its impact strength. These parameters are used to evaluate the mechanical contributions of each constituent material within the composite formulation.

**Table 7:** Datasets for ANN prediction.

Model	Sequence number	Inputs (20 random Wt.% of chosen components)	Outputs	Data source
Sequential ANN (composite model)	1	Flax fiber	Tensile strength, flexural strength, and impact strength	Synthesized via dirichlet distribution
		Pineapple leaf fiber		
		Epoxy		
		Coconut shell powder		
	2	Flax fiber	Tensile strength, flexural strength, and impact strength	
		Pineapple leaf fiber		
		Epoxy		
		Teak wood dust powder		
	3	Flax fiber	Tensile strength, flexural strength, and impact strength	
		Pineapple leaf fiber		
		Epoxy		
		Eggshell powder		
	4	Flax Fiber	Tensile strength, flexural strength, and impact strength	
		Pineapple leaf fiber		
		Epoxy		
		Rice husk powder		
	5	Flax fiber	Tensile Strength, Flexural Strength, and Impact Strength	
		Pineapple leaf fiber		
		Epoxy		

The formulated ANN model consists of an input layer includes an input layer with four neurons corresponding to the four constituent materials, followed by two hidden layers—the first with 16 neurons using ReLU activation and the second with 8 neurons. A single-neuron output layer is used to predict either Tensile Strength, Flexural Strength, or Impact Strength. The model is trained using the Adam optimizer with Mean Squared Error (MSE) as the loss function. It is trained over 50 epochs using validation data, with the test dataset containing predefined material weight compositions. After training, the model predicts the strength properties for these compositions, and the predicted results are compared with experimental values. The absolute percentage error is calculated using Eqs. (14), (15), and (16), respectively, and the prediction accuracy is evaluated using Eqs. (17), (18), and (19).

14 $$\:{APE}_{TS}=\frac{\left|{TS}_{exp}-{TS}_{pre}\right|}{{TS}_{exp}}\times\:100$$

15 $$\:{APE}_{FS}=\frac{\left|{FS}_{exp}-{FS}_{pre}\right|}{{FS}_{exp}}\times\:100$$

16 $$\:{APE}_{IS}=\frac{\left|{IS}_{exp}-{IS}_{pre}\right|}{{IS}_{exp}}\times\:100$$

Where, TS_exp_ refers to the experimentally measured tensile strength, while TS_pre_ represents the tensile strength predicted by the model, the accuracy of tensile strength prediction is determined using Eq. (17). Similarly, FS_exp_ and FS_pre_ denote the experimental and predicted values of flexural strength, respectively, with their prediction accuracy calculated using Eq. (18). IS_exp_ stands for the experimental impact strength, and IS_pre_ indicates the corresponding predicted impact strength, whose accuracy is evaluated according to Eq. (19).

17 $$\:{Accuracy}_{TS}=100-{APE}_{TS}$$

18 $$\:{Accuracy}_{FS}=100-{APE}_{FS}$$

19 $$\:{Accuracy}_{IS}=100-{APE}_{IS}$$

## Results and discussion

### Tensile test results

**Fig. 5:**
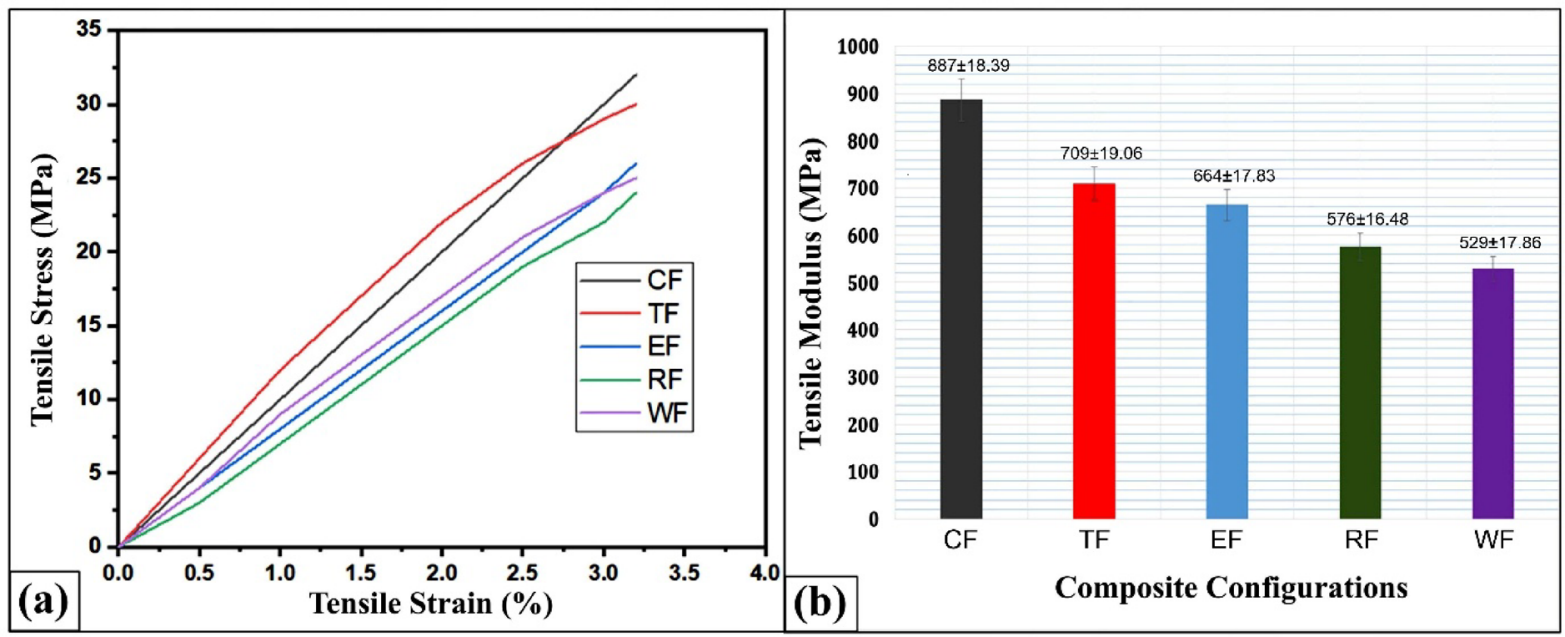
(**a**) Tensile stress–strain curves for different flax-pineapple-epoxy hybrid composites; (**b**) Variation of tensile modulus across composite configurations.

Figure 5a presents the stress–strain curves of various flax–pineapple–epoxy hybrid composites, clearly exhibiting a brittle fracture behavior across all configurations. This is evidenced by the absence of a yield point, indicating limited plastic deformation prior to failure, which is typical of thermoset-based composites. Among all composite types studied, the CSP (coconut shell powder)-reinforced composite exhibits the highest ultimate tensile strength, outperforming the WF, RHP, ESP, and TWD composites by 35.35%, 30.99%, 25.83%, and 19.13%, respectively. Similarly, Fig. 5b shows the tensile modulus of the same composite configurations. Again, CSP-based composites demonstrate the best performance, surpassing the tensile modulus values of WF, RHP, ESP, and TWD by 40.36%, 35.06%, 25.14%, and 20.06%, respectively. These significant improvements are attributed to the uniform dispersion of coconut shell powder within the epoxy matrix and its chemical compatibility with the matrix material.

Coconut shell particles are rich in cellulose and lignin, and the presence of abundant hydroxyl (-OH) groups enhances hydrogen bonding and chemical affinity with the epoxy matrix. These interactions result in improved interfacial adhesion and more efficient stress transfer during mechanical loading. Previous findings by Badyankal et al.^45^ and Sanadi et al.^46^ support this claim, indicating that such natural fillers act as effective reinforcements, particularly when finely ground and uniformly distributed. As a result, CSP-reinforced composites are promising for regular engineering applications^47^.

The TWD (teak wood dust)-based composite also shows reasonably high tensile performance, albeit slightly inferior to CSP. This behavior is attributed to the nucleating behavior of teak wood dust, which alters the crystallization pattern of the epoxy resin and somewhat disrupts the continuity of the fiber–matrix interface^48,49^. While TWD promotes stiffness, its interaction with the matrix is less chemically favorable compared to CSP, resulting in modest improvements in tensile strength. In the case of WF (wood flour) composites, tensile properties decrease noticeably, mainly due to poor fiber dispersion, void formation, and dominant fiber–fiber interactions. Such structural issues hinder effective load transfer and increase stress concentration points. These findings align with observations by Ashori et al.^50^, who noted similar drawbacks in rice straw–polypropylene composites. For the ESP (eggshell powder) composites, particle agglomeration is the main factor contributing to lower tensile strength and modulus. The inconsistent distribution of eggshell particles leads to weak interfacial zones, which fail to effectively transfer stress and become prone to early crack initiation and propagation under tensile loading^51^.

### Flexural test results

**Fig. 6:**
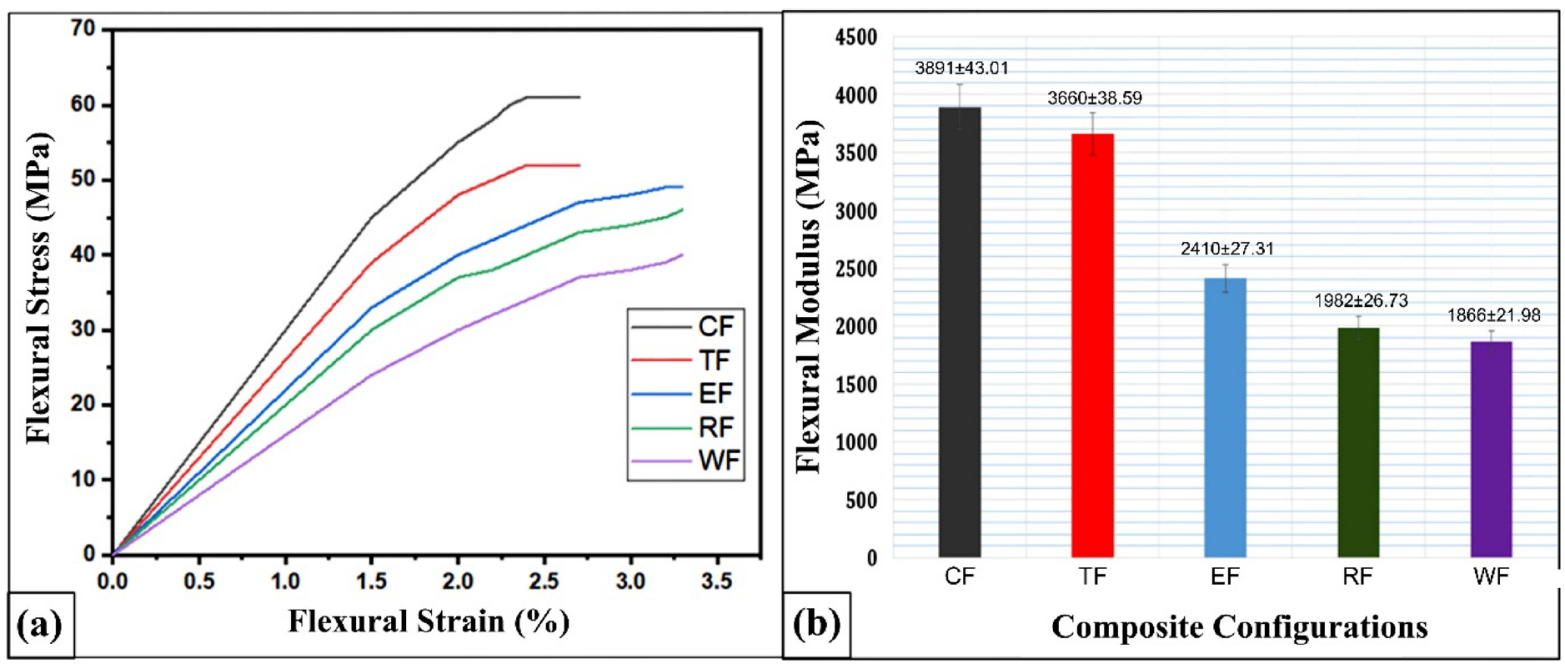
(**a**) Flexural stress–strain curves for different flax-pineapple-epoxy hybrid composites; (**b**) Comparison of flexural modulus among various composite configurations.

Figure 6a illustrates the flexural stress–strain curves for the developed flax–pineapple–epoxy hybrid composites. Among them, the CSP (Coconut Shell Powder) composite demonstrates a markedly superior flexural strength, outperforming the WF, RHP, ESP, and TWD composites by 35.67%, 21.42%, 17.98%, and 9.46%, respectively. Similarly, Fig. 6b shows that CSP composite also yields the highest flexural modulus, with enhancements of 52.04%, 49.06%, 38.06%, and 5.93% over the WF, RHP, ESP, and TWD composites, respectively.

This exceptional performance of the CSP composite is attributed to several factors. Firstly, the fine particle size and rigid nature of coconut shell powder provide better stress transfer during bending. More importantly, the hydroxyl (-OH) functional groups available on the surface of CSP facilitate strong hydrogen bonding with the polar functional groups in the epoxy matrix. CSP is rich in lignocellulosic components specifically cellulose, hemicellulose, and lignin which are known for enhancing the mechanical properties of polymer composites through intermolecular adhesion^52,53^. The presence of these natural biopolymers increases compatibility with epoxy matrices, leading to improved dispersion and a reduction in void formation. Furthermore, cellulose and lignin have an affinity for polar compounds like methylal and phenol, which can further enhance interfacial adhesion and stability under flexural loading conditions^52,53^.

In comparison, the TWD (Teak Wood Dust) composite exhibits comparatively lower flexural performance. This is largely due to the compact grain and oily nature of teak, which inhibits resin penetration and bonding efficiency within the composite. The naturally occurring silica and extractives in teak can also interfere with fiber-matrix adhesion, resulting in poor stress transfer and lower mechanical output^54^.

The ESP (Eggshell Powder) composite, while offering modest mechanical benefits, suffers from filler agglomeration, which creates localized stress concentration zones. These zones act as crack initiation sites and degrade the overall flexural behavior of the composite. The brittle nature and irregular geometry of eggshell particles also disrupt matrix continuity, weakening both strength and stiffness under flexural load^55^. The RHP composite also suffers from reduced flexural strength, primarily due to poor bonding between the rice husk powder and the epoxy resin^56^.

### Inter laminar shear strength test results

**Fig. 7:**
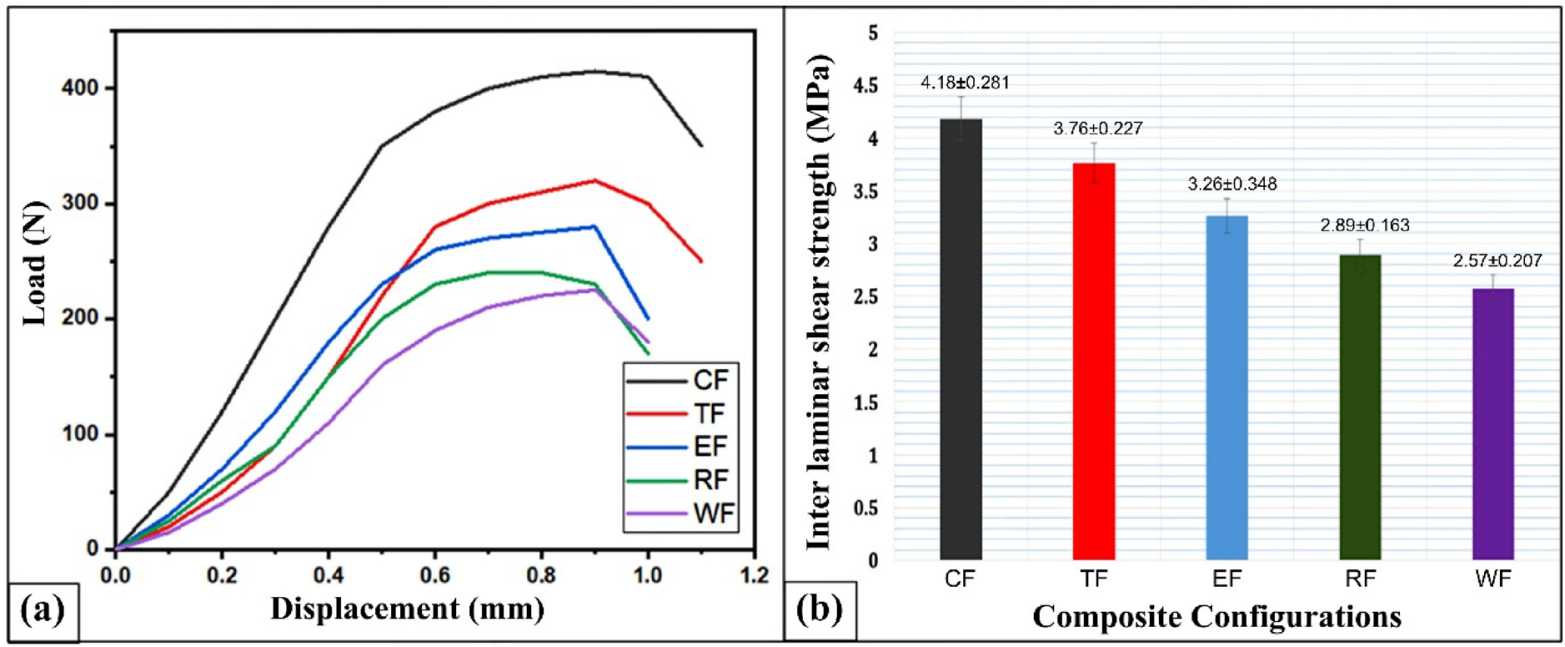
(**a**) Force–displacement curves and (**b**) Interlaminar shear strength (ILSS) values of different flax-pineapple-epoxy hybrid composites under ILSS testing.

Figure 7a shows the load-displacement curves for various composite types, revealing that the CSP (coconut shell powder) composite has the highest load-bearing capacity. Figure 7b presents the Interlaminar Shear Strength (ILSS) values, highlighting that the CSP composite surpasses the WF, RHP, ESP, and TWD variants by 38.51%, 30.86%, 22%, and 10.05%, respectively.

The superior ILSS performance of the CSP composite is primarily due to the uniform dispersion of finely milled coconut shell particles within the epoxy matrix, which fosters strong interfacial adhesion between the filler and resin. This uniform distribution minimizes micro-voids and enhances load transfer. The presence of hydroxyl (-OH) groups in CSP supports chemical bonding with the epoxy network, further reinforcing interfacial strength. Similar improvements in ILSS were reported by T.P. Sathishkumar and S. Ramakrishnan^57^ in jute mat-reinforced epoxy composites using nano-sized coconut shell fillers. Conversely, TWD composite increases ILSS because of the teak wood dust agglomeration and lack of random distribution inside the matrix that prevents the formation of more homogenous Composites with better structural performance.

Comparatively, the composite (TWD) of teak wood dust has a lower ILSS value, which is related to uneven distribution and agglomeration of teak particles into the matrix. This discrepancy presents regional defects and areas of insufficiency in shear load transfer. These findings coincide well with the findings by Maruthi Prashanth et al.^19^ in abaca-pineapple-epoxy composites, in which teak dust filler compromised interlaminar integrity. Clustering of eggshell particles also leads to a reduction in the ILSS performance of the ESP (eggshell powder) composite as it generates stress concentration points and areas of poor fiber-matrix interface, both of which hinder the effective dissipation of shear stress; similar trends have been reported in studies looking into ESP in fiber-filled matrices^58^. The RHP (rice husk powder) composite has moderate ILSS values, which is indicative of less than optimum interfacial bonding, perhaps due to the chemistry of surface material and deposits of silica-rich husk particles that interfere with the hold of the matrix, causing lower shear at load.

### Impact test results

**Fig. 8:**
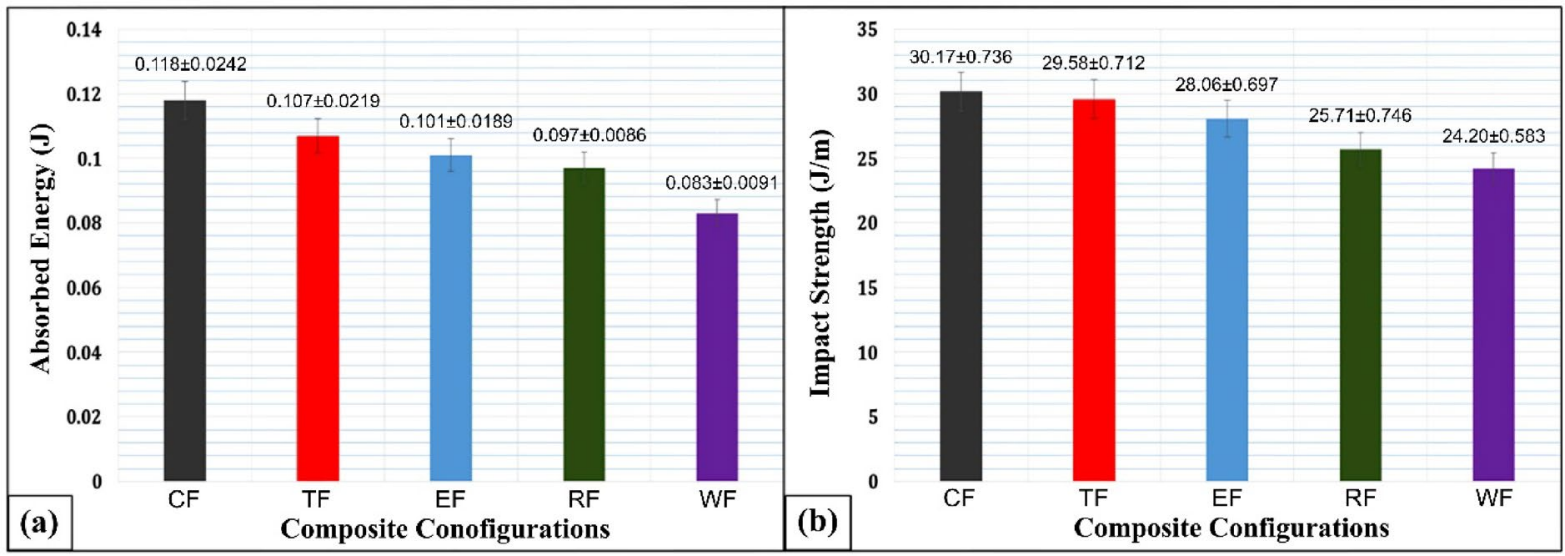
(**a**) Absorbed energy and (**b**) Impact strength of various flax-pineapple-epoxy hybrid composites across different configurations.

Figure 8a illustrates the energy absorption behavior under impact loading. The CSP (coconut shell powder) composite absorbs significantly more energy—29.66%, 17.79%, 14.40%, and 9.32% more than the WF, RHP, ESP, and TWD composites, respectively. In Fig. 8b, the impact strength of CSP composite also leads, outperforming WF by 19.78%, RHP by 14.78%, ESP by 6.99%, and TWD by 1.95%.

**Fig. 9:**
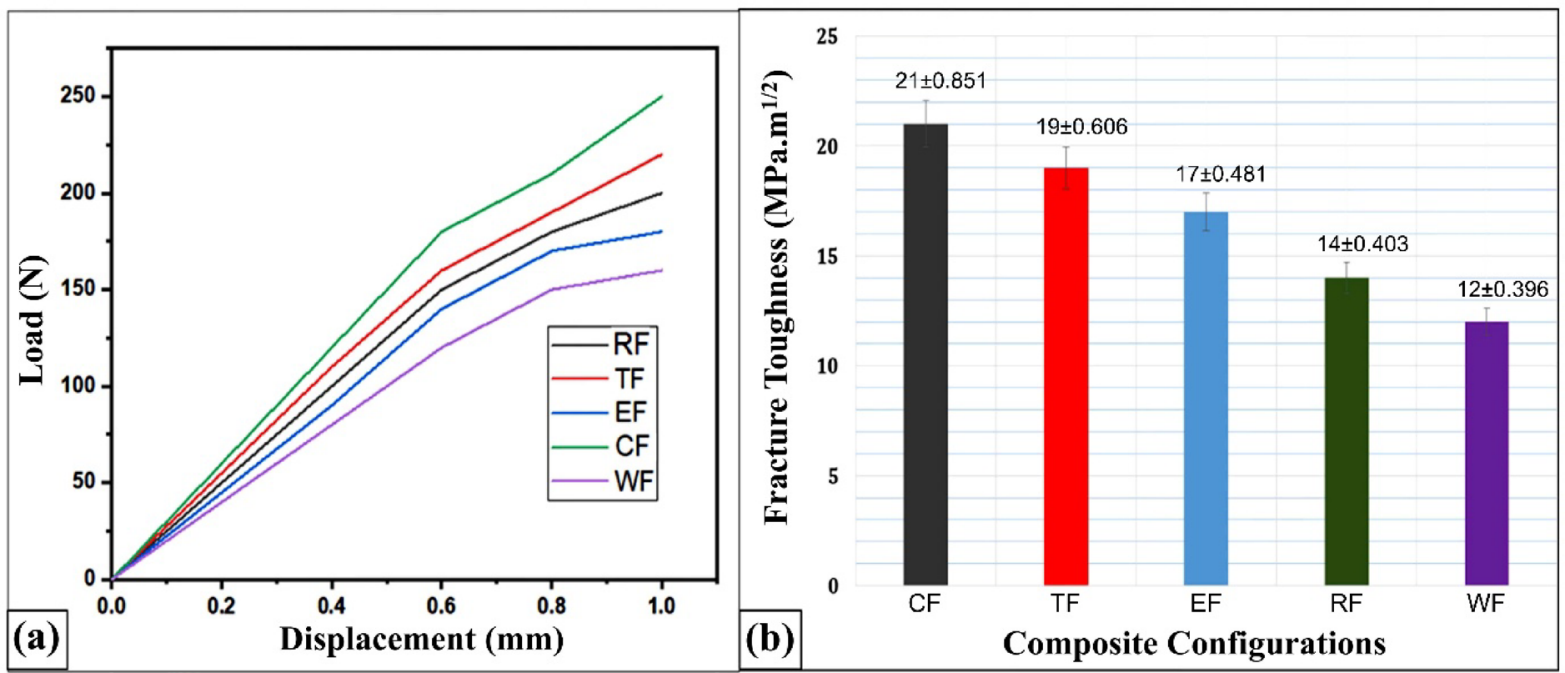
Micro-SEM images of fractured composite samples under load: (**a**) CSP, (**b**) TWD, (**c**) ESP, and (**d**) RHP.

The impact performance of a composite largely depends on the toughness of the reinforcing material, the quality of the interface between matrix and filler, and the amount of frictional energy required to dislodge fillers. The strength of this interfacial region is critical and directly influences the composite’s ability to absorb and dissipate impact energy^59^. As evidenced in Fig. 9, the superior impact performance of CSP composites results from multiple synergistic factors - Energy-dissipating microstructure, CSP particles have a lignocellulosic structure that can deform and absorb energy before fracture. Strong filler–matrix adhesion, Surface hydroxyl groups in CSP facilitate bonding with the epoxy resin, enhancing stress transmission at the interface. These combined effects allow CSP composites to absorb and dissipate more impact energy efficiently consistent with findings in coconut shell particulate-filled composites, which showed improved impact resistance in aggressive environments.

In comparison, TWD composites exhibit lower impact performance due to weaker filler–matrix bonding and limited energy dissipation, likely caused by teak particles’ dense grain and poor wetting. This aligns with observations that teak-filled hybrid composites often show reduced interfacial energy transference and brittleness under impact loading. For the ESP composite, impact strength is hindered by particle agglomeration and microstructural inhomogeneities, creating stress concentration zones that lead to premature crack initiation—similar to results observed in eggshell/sisal hybrid composites where excessive filler loads reduced impact toughness. Comparable results were reported by Oladele et al.^60^ for eggshell/sisal hybrid epoxy composites, reinforcing the effect of filler incompatibility on mechanical behavior. The RHP composite also shows moderate performance, linked to inadequate dispersion and chemical compatibility of rice husk fillers, which reduces energy absorption capacity when stressed. Such composites often exhibit brittle fracture behavior due to ineffective stress transfer through the matrix.

### Single edge notch bending (SENB) test results

**Fig. 10:**
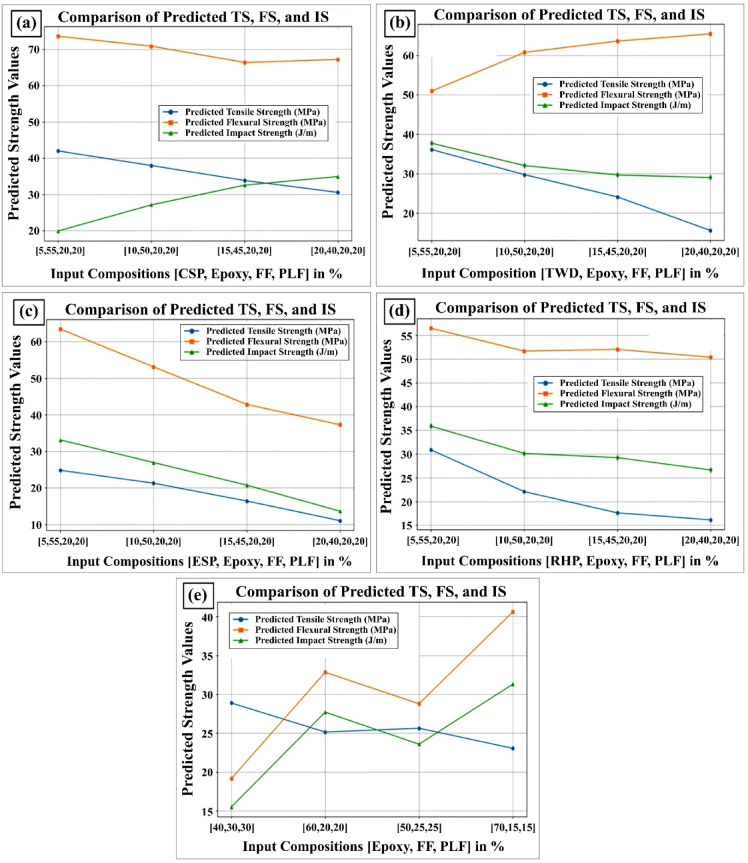
(**a**) Load–deformation curves and (**b**) Fracture toughness of various flax-pineapple-epoxy hybrid composites.

Figures 10a and 9b display the load–displacement behavior and corresponding mode-I fracture toughness (K_IC_) values for the various flax–pineapple–epoxy hybrid composite configurations. Among the tested samples, the Coconut Shell Powder (CSP)-filled composite exhibited the highest load-bearing capacity and fracture resistance. Quantitatively, the fracture toughness of the CSP composite was enhanced by 42.8%, 33.3%, 19.04%, and 9.52% compared to the WF (without filler), RHP (Rice Husk Powder), ESP (Eggshell Powder), and TWD (Teak Wood Dust) composites, respectively.

The superior fracture toughness of the CSP composite can be primarily attributed to the rigid microstructure and high stiffness of coconut shell particles, which act as micro-barriers to crack initiation and growth. These particles promote effective energy dissipation mechanisms such as crack deflection, crack bridging, and matrix shear yielding, which increase the resistance to crack propagation. Furthermore, the strong interfacial adhesion between the CSP particles and the epoxy matrix enhanced by the presence of hydroxyl functional groups contributes to improved load transfer and crack-arresting capability under applied stress.

The toughness behavior is also influenced by the homogeneous dispersion of CSP particles within the matrix. Uniform distribution minimizes void formation and eliminates premature failure zones, ensuring that cracks encounter consistent resistance during propagation.

In contrast, the TWD-filled composite exhibits a comparatively lower fracture toughness. This reduction can be attributed to the interlocking grain structure of teak wood dust and its poor interfacial compatibility with the epoxy matrix. The result is the formation of localized stress concentration zones, where the filler matrix interaction is not strong enough to prevent crack initiation. Under load, these zones experience stress intensification, leading to crack propagation before significant energy can be dissipated through toughening mechanisms^61^.

The RHP composite also suffers from reduced toughness, primarily due to agglomeration of rice husk particles. This agglomeration leads to heterogeneous regions within the matrix, interrupting the uniform stress field and reducing the crack resistance. Additionally, the inherent silica content of rice husk creates a weak interface with the organic polymer matrix, resulting in debonding under stress, which further lowers fracture resistance. Similar interfacial debonding mechanisms were reported by Ashori, Alireza^50^. in rice-based composite systems.

The ESP composite, while offering moderate performance, is limited by the brittle nature of the eggshell filler and incompatibility in stiffness between the filler and matrix. These mismatches reduce the matrix’s ability to redistribute stress effectively, allowing cracks to propagate more easily through the weak interfacial regions. Table 8 shows the mechanical properties of different composite configurations with mean values and standard errors.

**Table 8:** Mechanical properties of developed composite.

Composite	Tensile modulus (MPa)	Tensile strength (MPa)	Flexural strength MPa	Flexural modulus (MPa)	Fracture toughness (MPa·m^1/2^)	ILSS (MPa)	Absorbed energy (J)	Impact strength (J/m)
CF	887 ± 18.39	33 ± 1.03	62 ± 2.51	3891 ± 43.01	21 ± 0.851	4.18 ± 0.281	0.118 ± 0.0242	30.17 ± 0.736
TF	709 ± 19.06	30 ± 1.87	52 ± 1.54	3660 ± 38.59	19 ± 0.606	3.76 ± 0.227	0.107 ± 0.0219	29.58 ± 0.712
EF	664 ± 17.83	26 ± 2.08	47 ± 1.8	2410 ± 27.31	17 ± 0.481	3.26 ± 0.348	0.101 ± 0.0189	28.06 ± 0.697
RF	576 ± 16.48	24 ± 0.98	43 ± 1.3	1982 ± 26.73	14 ± 0.403	2.89 ± 0.163	0.097 ± 0.0086	25.71 ± 0.746
WF	529 ± 17.87	23 ± 1.24	38 ± 2.01	1866 ± 21.98	12 ± 0.396	2.57 ± 0.207	0.083 ± 0.0091	24.20 ± 0.583

### Machine learning model results

The Artificial Neural Network (ANN) model was developed using a dataset of 20 composite formulations for training. Network weights were optimized through iterative adjustment until high predictive accuracy was achieved, as evidenced by strong alignment between model outputs and experimental measurements. ANNs are particularly well-suited for capturing material responses characterized by complex nonlinear interactions among multiple input factors—such as filler content, fiber types, and processing conditions—making them a robust choice for modeling composite behavior^62^.

A sensitivity analysis of the input features revealed that the weight% of the filler consistently exerted the greatest influence on predicted tensile, flexural, and impact strengths, underscoring its primary role in determining mechanical performance across hybrid composite configurations.

**Fig. 11:**
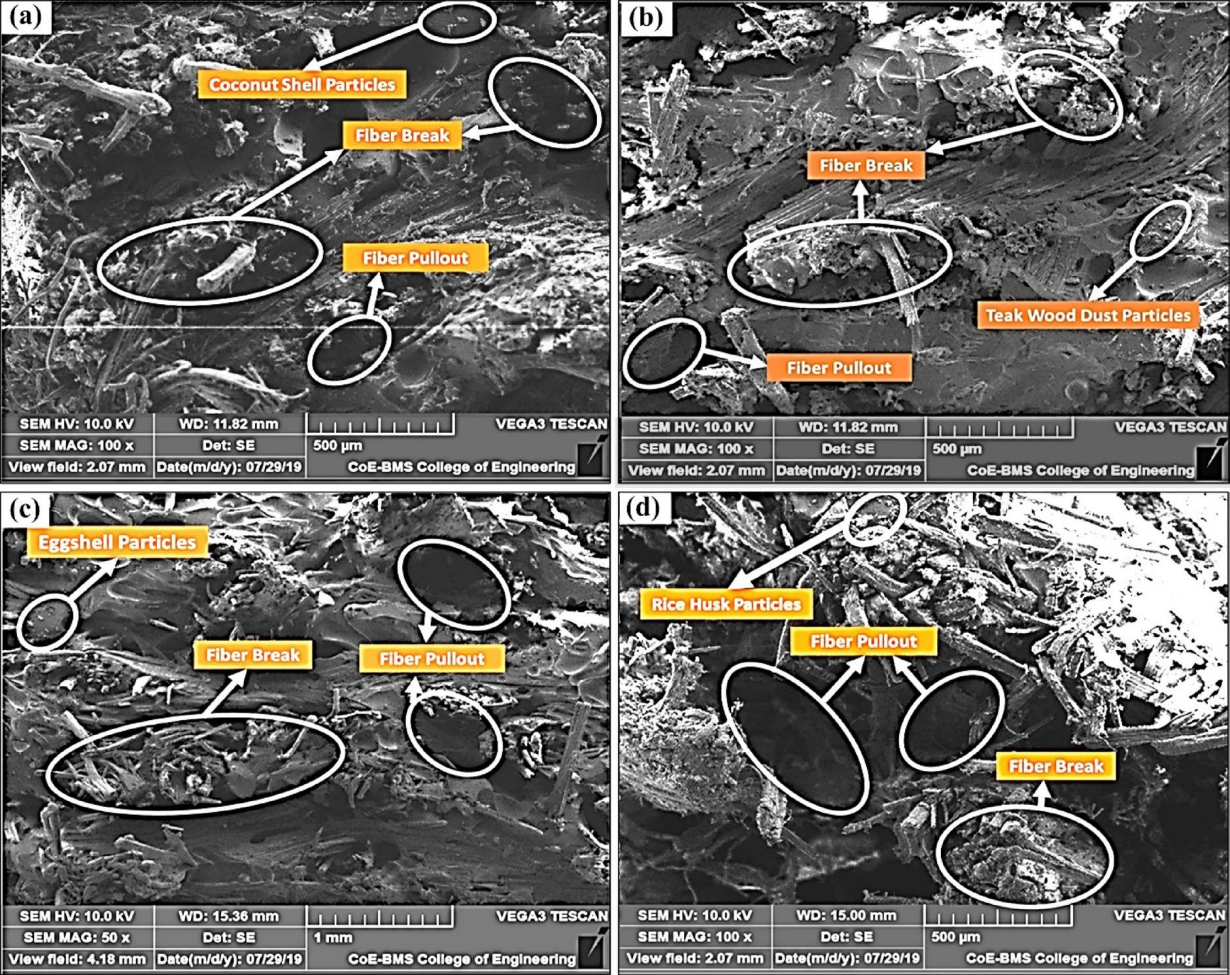
Predicted vs. experimental results of ANN-based tensile strength, flexural strength, and impact strength for flax-pineapple-epoxy hybrid composites with different bio-waste fillers: (**a**) CSP (coconut shell powder), (**b**) TWD (teak wood dust), (**c**) ESP (eggshell powder), (**d**) RHP (rice husk powder), and (**e**) WF (without filler) configurations.

Figure 11a–e compare the predicted versus measured values for each mechanical property across all composite formulations. The ANN model demonstrated exceptionally high accuracy:

- Tensile strength accuracies: CSP 95.85%, TWD 99.16%, ESP 78.83%, RHP 87.73%, WF 93.22%.
- Flexural strength accuracies: CSP 83.9%, TWD 90.01%, ESP 93.83%, RHP 92.18%, WF 83.7%.
- Impact strength accuracies: CSP 89.83%, TWD 91.78%, ESP 96.01%, RHP 82.85%, WF 85.48%.

Corresponding absolute percentage errors (APE) were small, ranging from 0.84% to 21.17%:

- Tensile APE: CSP 4.15%, TWD 0.84%, ESP 21.17%, RHP 12.27%, WF 6.78%.
- Flexural APE: CSP 16.1%, TWD 9.99%, ESP 6.17%, RHP 7.82%, WF 16.3%.
- Impact APE: CSP 10.17%, TWD 8.22%, ESP 3.99%, RHP 17.15%, WF 14.52%.

These results align with regression curves and correlation coefficients (R² > 0.96 for tensile and flexural predictions found in similar studies by Cui et al.^62^, L. Natrayan^63^, confirming the ANN’s capability to generalize well even for small datasets.

The model’s performance—particularly the influence of filler percentage—mirrors findings from polymer composite research, where ANNs effectively predict mechanical behavior based on filler concentration and morphology^63^, Additionally, the observed prediction errors are within the ranges reported by similar ANN studies, e.g., composite strength estimates typically achieve > 95% accuracy^62,63^.

Overall, this ANN model demonstrates that with proper training and optimization, even a modestly sized dataset of 20 samples can yield highly accurate predictions of key mechanical properties. This approach represents a time-saving, cost-effective methodology for composite design, enabling rapid performance evaluation without extensive experimental testing—a trend increasingly documented in modern materials science, especially in additive manufacturing and natural-fiber composites^62,63^.

### SEM analysis

The Scanning Electron Microscopy (SEM) analysis provided valuable insights into the fractured surfaces of various flax-pineapple-epoxy hybrid composites filled with different bio-waste materials. Figure 9a shows the CSP configuration, where coconut shell particles exhibit strong interfacial adhesion with the epoxy matrix, contributing to improved mechanical strength. In contrast, Fig. 9b reveals that the TWD composite displays significant fiber pull-out, indicating weak bonding between teak wood dust particles and the matrix, thereby reducing strength similar results were obtained by Sari et al.^64^ for corn husk fiber-epoxy composites.

Figure 9c highlights the ESP composite, where the agglomeration of eggshell powder disrupts uniform distribution, resulting in lower mechanical performance. Similarly, Fig. 9d illustrates the RHP configuration, where both agglomeration and fiber pull-out of rice husk particles were observed, leading to poor interfacial bonding and a corresponding decline in overall mechanical properties^65^.

## Applications

The developed composites, particularly those filled with coconut shell powder, exhibit enhanced tensile, flexural, and impact strengths, making them highly suitable for lightweight structural applications where a high strength-to-weight ratio is essential. The incorporation of natural fibers and bio-waste fillers enhances their sustainability, making them ideal for producing eco-friendly consumer goods such as furniture, travel luggage, and everyday utility items. Such composites with their mechanical robustness, biodegradability, and light weights are also apt to components of automobiles such as interior panels, dashboards, door trims, and other compositions that make the automobile sturdy. Within the construction industry, they can be used to make wall panels, ceilings boards, partitions and decor elements because of their moderate strength and environmental advantages. Moreover, the property of durability, strength, and less weight make them applicable in the production of sporting products such as sporting helmets, protective accessories, and sporting light weights. Finally, the composites offer a lasting substitute to electronic encasings and panels- both structural durability and environmentally friendly appearance forms important design aspects.

## Conclusion

In this study, epoxy reinforced hybrid composites in which flax and pineapple leaf fibers were used in the manufacture process through hand lay-up method and then hot-pressed to provide good compacting and curing were done successfully. The study systematically investigated the influence of four bio-waste fillers—coconut shell powder (CSP), teak wood dust (TWP), eggshell powder (ESP), and rice husk powder (RHP)—each incorporated at 10 wt% to evaluate their effect on the composites’ mechanical behavior.

- Among all tested combinations, CSP-filled composites exhibited the highest and most consistent enhancement in mechanical performance, when compared to the filler-free (WF) control samples.
- The tensile strength and modulus of CSP composites increased by approximately 35% and 40%, respectively, confirming the effectiveness of CSP in improving stress transfer and strengthening fiber–matrix adhesion.
- Flexural strength and modulus were enhanced by 36% and 52%, while interlaminar shear strength (ILSS) rose by 39%, primarily due to uniform CSP dispersion and improved mechanical interlocking within the epoxy matrix.
- Impact tests revealed a 30% increase in absorbed energy and a 20% rise in impact strength, demonstrating the superior energy absorption capability of CSP-reinforced laminates compared to other filler systems.
- The fracture toughness, measured using the single-edge notch bending (SENB) test, improved by 42%, reflecting enhanced resistance to crack initiation and propagation resulting from the denser microstructure created by CSP addition.
- An Artificial Neural Network (ANN) model, developed using a Sequential approach, demonstrated high prediction accuracy—95.85% for tensile behavior, 83.9% for flexural properties, and 89.83% for impact responses. The strong agreement between ANN predictions and experimental data confirms its reliability and future applicability in composite property forecasting.
- Scanning Electron Microscopy (SEM) further confirmed superior interfacial bonding, reduced void formation, and more cohesive fracture surfaces in CSP composites, supporting the observed mechanical improvements.

This study contributes to the advancement of sustainable composite technology by not only optimizing bio-waste filler content in hybrid fiber-reinforced systems but also demonstrating the practical value of integrating ANN-based predictive modeling for intelligent and efficient material design. From an engineering perspective, the substantial gains in tensile, flexural, interlaminar, and fracture properties position CSP-reinforced natural fiber composites as promising candidates for lightweight structural panels, automotive interior parts, building elements, and environmentally sustainable consumer products.

However, the study is limited by the use of a single filler loading (10 wt%), the absence of long-term durability assessments (moisture, thermal aging, UV exposure), and the relatively small dataset used for ANN training, which may restrict model generalizability. Addressing these limitations in future studies will further enhance the development and industrial transition of high-performance, sustainable biocomposites.

## Possibilities and future work

Future research in this area holds significant potential for expansion. Upcoming studies can focus on how different particle sizes and weight proportions of bio-waste fillers, like eggshell powder, wood dust, and other agricultural by-products, affect the mechanical performance of the composites. Investigating how these composites behave under varying environmental conditions, such as humidity, temperature changes, and UV exposure, would help in understanding their long-term reliability. There is also a scope to refine processing parameters, including curing time, temperature, and applied pressure, to improve the overall material characteristics. Utilizing advanced evaluation methods such as dynamic mechanical analysis (DMA) and thermal degradation assessments can offer deeper insights into the behavior and stability of these materials. Additionally, examining their biodegradability and conducting life cycle analyses would contribute to promoting sustainable applications across industries.

## Data Availability

All data that support the findings of this study are included within the article.
